# Long-Term Outcomes of Bioprosthetic or Mechanical Valve Replacement in End-Stage Renal Disease: A Nationwide Population-Based Retrospective Study

**DOI:** 10.3389/fcvm.2021.745370

**Published:** 2021-12-17

**Authors:** Guan-Yi Li, Yun-Yu Chen, Fa-Po Chung, Kuo-Liong Chien, Chiao-Po Hsu, Yenn-Jiang Lin

**Affiliations:** ^1^Division of Cardiology, Department of Medicine, Taipei Veterans General Hospital, Taipei, Taiwan; ^2^Institute of Epidemiology and Preventive Medicine College of Public Health, National Taiwan University, Taipei, Taiwan; ^3^Cardiovascular Research Center, Institute of Clinical Medicine, National Yang-Ming University, Taipei, Taiwan; ^4^Division of Cardiovascular Surgery, Department of Surgery, Taipei Veterans General Hospital, Taipei, Taiwan

**Keywords:** bioprosthetic valve, cardiovascular event, end-stage renal disease, valve replacement, mechanical valve, mortality

## Abstract

**Background:** Valve replacement is associated with worse outcomes in individuals who have end-stage renal disease (ESRD) and require a long-term renal replacement therapy. Prosthetic valve selection in patients with ESRD has remained controversial.

**Objective:** We aimed to investigate long-term outcomes of mechanical and bioprosthetic valve replacement in individuals with ESRD.

**Methods:** We conducted a population-based retrospective cohort study using data obtained from the Taiwan National Health Insurance Research Database. In total, 10,202 patients, including 912 ESRD and 9,290 non-ESRD patients, were selected after a 1:1 propensity-score matching based on the type of prosthetic valve used. The long-term mortality outcomes were then analyzed.

**Results:** During a median follow-up period of 59.6 months, the Kaplan–Meier survival analysis revealed that ESRD patients who underwent mechanical valve replacement had higher rates of all-cause mortality and CV deaths than those who underwent bioprosthetic valve replacement (Log-rank test, *p* = 0.03 and 0.02, respectively). Multivariable regression analyses demonstrated that ESRD patients who underwent bioprosthetic valve replacement had lower rates of all-cause mortality (*p* < 0.001, hazard ratio: 0.88, 95% confidence interval: 0.82–0.93) and cardiovascular (CV) death (*p* < 0.001, hazard ratio: 0.83, 95% confidence interval: 0.76–0.90) than those who had mechanical valve replacement.

**Conclusion:** Bioprosthetic valve replacement is significantly associated with lower rates of all-cause mortality and CV death in the ESRD population.

## Introduction

Choosing a prosthetic heart valve can be clinically challenging, and it is commonly based on several factors, such as age, underlying disease requiring the use of anticoagulants, risk of bleeding and thromboembolism, durability of the prosthesis, patients' preferences, and risk of structural deterioration requiring re-interventions (1, 2). Of note, the type of valve prosthesis that should be used in a specific population with comorbidity, including end-stage renal disease (ESRD), has been debated for decades (3–7).

It has long been established that the abnormal calcium and phosphate metabolism due to ESRD is related to calcification and degenerative valvular lesions, which may be explained by an active regulated process associated with an osteoblast-like phenotype (8–11). It results in a major concern regarding structural destruction of the bioprosthetic valves in ESRD. Thus, mechanical valves were previously recommended for ESRD patients (12).

In contrast, patients with ESRD receiving anticoagulants are at higher risk of bleeding, (13, 14) metastatic calcification, and even calciphylaxis (15, 16). In addition, ESRD patients have a short life expectancy. As a result, the increased durability of a mechanical valve may only benefit a small portion of ESRD patients (17–22).

The number of studies investigating the clinical outcome between dialysis patients who had bioprosthetic and mechanical valve replacement is increasing worldwide (23–25). However, owing to the limited sample size, non-uniform characteristics, and emerging advancements in prosthesis and clinical care of dialysis patients, previous studies had conflicting results, and some have shown a similar survival between dialysis patients who had mechanical and bioprosthetic valve replacement (26–30). Thus, the abovementioned findings were not validated in a large-scale nationwide population study. Furthermore, no specific recommendation regarding the selection of prosthetic valves for patients with ESRD was provided in the contemporary guideline (31).

This nationwide population-based study aimed to assess long-term outcomes and associated cardiovascular (CV) events in ESRD patients who underwent bioprosthetic and mechanical valve replacement. We believe that the abovementioned findings could provide insight on decision-making regarding the selection of prosthetic valves among ESRD patients.

## Materials and Methods

### Study Design and Participants

We conducted a population-based retrospective cohort study, and data were collected from January 1, 2000 to December 31, 2011. The patients who underwent the first valve replacement surgery, without previous or concomitant valve repair, were identified using information from the National Health Insurance Research Database (NHIRD), and they were grouped based on the procedure code of the Specifications of the National Voluntary Consensus Standards for Cardiac Surgery: bioprosthetic valve replacement (procedure code: 35.21, 35.23, 35.25, and 35.27) and mechanical valve replacement (procedure code: 35.22, 35.24, 35.26, and 35.28).

This study was approved in accordance with the Good Clinical Practice Guidelines by the Research Ethics Committee C of the National Taiwan University Hospital.

### Databases and Specifications of the Characteristics of the Participants

The Taiwan Collaboration Center of Health Information Application, Ministry of Health and Welfare, provided all the datasets of the NHIRD. The Taiwan's National Health Insurance (NHI) program enrolled 23 million people, which covered 99% of the country's population and included utilization of all NHI resources, including outpatient visits, hospital care, prescribed medications, and the National Death Registry. We obtained permission for the rights from the National Research Institute for the Department of Health and the Health Promotion Administration, Ministry of Health and Welfare. The underlying diseases were identified according to the International Classification of Diseases, 9^th^ Revision—Clinical Modification (ICD 9-CM) codes. The diagnosis must be recorded twice in the outpatient records or at least once in the inpatient records. By linking to the NHIRD, we identified clinical variables, such as age (years), sex, type of valve replacement, number of valve replacements, and presence of chronic kidney disease, congestive heart failure, acute coronary diseases, chronic obstructive pulmonary disease, and thyroid diseases. The selection and grouping of medications were based on the guidelines of the Anatomical Therapeutic Chemical classification system by the World Health Organization.

Patients diagnosed with ESRD were identified using the order codes (Supplementary Material) of hemodialysis, peritoneal dialysis, and other types of dialysis for at least 3 months (32). In this study, we excluded individuals who were younger than 20 years, underwent both bioprosthetic and mechanical valve replacement, or presented with lethal ventricular arrhythmias before the procedures. In addition, no patient underwent kidney transplantation prior to enrollment.

### Study Endpoints During the Long-Term Follow-Up

The primary endpoints were all-cause mortality and CV death (ICD 9-CM codes 390–429) during follow-up. Death was confirmed using data from the Taiwan's National Death Registry. Follow-up was terminated in case of death or if the patients lived beyond December 31, 2016.

### Statistical Analysis

The normally distributed continuous variables are presented as mean values ± standard deviation, and non-normally distributed continuous variables are presented as medians with 25 and 75% interquartile ranges (IQRs). Student's *T*-test was utilized to compare two groups. For testing the distribution of general continuous variable such as age, the normality test was performed before the Student's *T*-test. Categorical variables were expressed as numbers and percentages and were compared using the chi-square test. The incidence rates of CV events were calculated as the number of cases per 1,000 person-years during follow-up. Propensity-score matching for patients receiving the mechanical valve replacement and the bioprosthetic valve replacement as exposures were performed to minimize the impact of the confounding factors on the clinical outcomes, including age, sex, hypertension, diabetes mellitus, chronic obstructive pulmonary disease, congestive heart failure, stroke, and total number of valves replaced. A one-to-one matching of pairs was conducted using identical propensity scores with a 0.15 caliper width.

The event-free survival curve was plotted using the Kaplan–Meier method with the statistical significance examined using the Log-rank test. The conditional Cox proportional-hazards regression model was utilized to compare the hazard ratios (HRs), with 95% confidence intervals (CIs), of the outcomes. The potential confounders were adjusted using three models (Model 1: age and sex; Model 2: Model 1 plus total number of valves replaced, hypertension, diabetes mellitus, congestive heart failure, coronary artery diseases, and chronic obstructive pulmonary disease; and Model 3: Model 2 plus the use of medications (antiarrhythmic agents of Ia Ib, Ic, III, calcium channel blockers, angiotensin receptor blockers, statins, insulin, and oral hypoglycemic agents). The level of statistical significance was set at a two-tailed alpha level <0.05. The analyses were performed with SAS software (version 9.4, SAS Institute, Cary, NC).

## Results

### Selection and Characteristics of the Study Population

**Figure 1:**
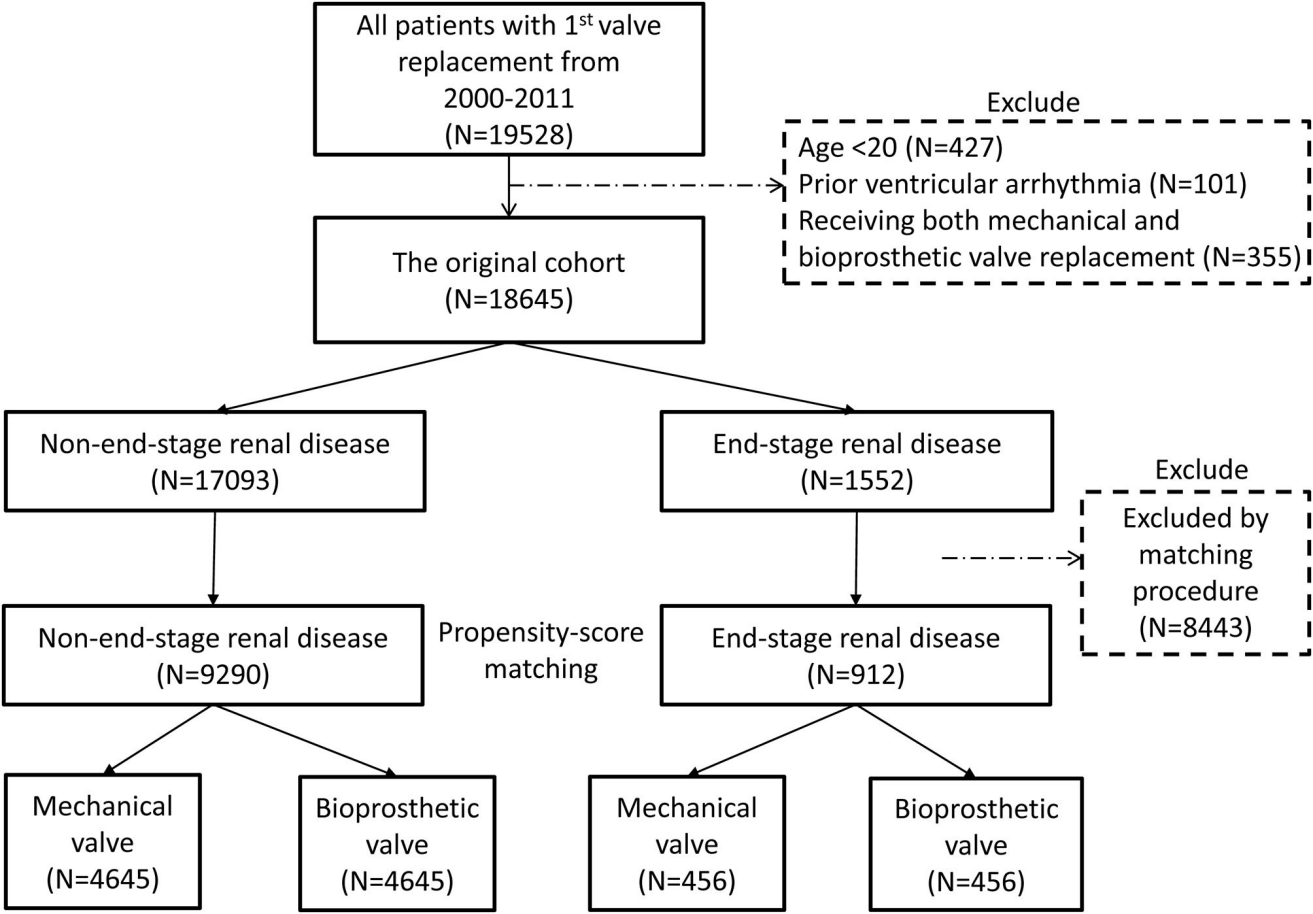
Flow chart of the present study. The process of study population selection and propensity score matching is presented. After PSM, 9,290 and 912 patients were included in the non-ESRD group and ESRD group, respectively. Both groups had equal number of patients who had mechanical and bioprosthetic valve replacement. ESRD, end-stage renal disease; PSM, propensity score matching.

In total, 19,528 patients who had their first valve replacement were identified in the NHIRD. After excluding 883 patients according to the exclusion criteria, 18,645 were included in the original cohort (Supplementary Table 1), and 10,202 patients were selected after propensity-score matching (PSM) (Supplementary Table 2). After PSM, 9,290 and 912 patients were included in the non-ESRD group and ESRD group, respectively. Both groups had equal number of patients who had mechanical and bioprosthetic valve replacement (Figure 1, Table 1, Supplementary Table 3).

**Table 1:** Baseline characteristics of ESRD cohorts after propensity-score matching.

**Variables**	**ESRD group** (***N*** **= 912)**		
	**Mechanical valve** (***N*** **= 456)**	**Bioprosthetic valve** (***N*** **= 456)**	***P*** **value**
**Age**	67.4 ± 11.8	66.8 ± 11.9	0.51
**Male gender**	240 (52.6%)	254 (55.7%)	0.35
**Valve location**			
Aortic valve	242 (53.1%)	234 (51.3%)	0.60
Mitral valve	246 (53.9%)	254 (55.7%)	0.60
Tricuspid valve	13 (2.9%)	27 (5.9%)	0.02
Pulmonary valve	0 (0%)	3 (0.7%)	0.24
**Total number of valves replaced**	1.10 ± 0.30	1.14 ± 0.35	0.08
1	411 (90.1%)	395 (86.6%)	0.18
2	45 (9.9%)	59 (12.9%)	
3	0 (0%)	2 (0.44%)	
4	0 (0%)	0 (0%)	
**Comorbidities**			
ESRD (%)	456 (100%)	456 (100%)	>0.99
Diabetes mellitus (%)	16 (3.5%)	22 (4.8%)	0.32
Hypertension (%)	53 (11.6%)	62 (13.6%)	0.37
COPD (%)	4 (0.9%)	4 (0.9%)	>0.99
CHF (%)	118 (25.9%)	110 (24.1%)	0.54
Prior stroke (%)	16 (3.5%)	22 (4.8%)	0.32
Prior CAD (%)	53 (11.6%)	24 (5.3%)	0.001
Thyroid disease (%)	0 (0%)	0 (0%)	>0.99
Pharmacotherapy^*^			
**AADs (%)**	146 (32%)	175 (38.4%)	0.001
Class Ia	7 (1.5%)	8 (1.8%)	0.80
Class Ib	35 (7.7%)	42 (9.2%)	0.40
Class Ic	24 (5.3%)	29 (6.4%)	0.48
Class III	107 (23.5%)	138 (30.3%)	0.02
CCB (%)	203 (44.5%)	196 (42%)	0.64
ARB (%)	274 (60.1%)	322 (70.6%)	0.001
Statins (%)	184 (40.4%)	214 (46.9%)	0.045
Insulin (%)	134 (29.4%)	156 (34.2%)	0.12
OHA (%)	168 (36.8%)	192 (42.1%)	0.10

* *Used from baseline till the end of follow-up*.

*AAD, antiarrhythmic drugs; ARB, angiotensin receptor blockers; CAD, coronary artery disease; CCB, calcium channel blocker; CHF, congestive heart failure; COPD, chronic obstructive pulmonary disease; ESRD, end-stage renal disease; OHA, oral hypoglycemic agents*.

After PSM, a higher number of patients in the ESRD group underwent bioprosthetic valve replacement for the tricuspid valve (5.9 vs. 2.9%, *p* = 0.02; Table 1). In the ESRD group, the baseline characteristics were comparable between patients who had mechanical and bioprosthetic valve replacement, except that a high number of patients with mechanical valve replacement had a history of coronary artery disease (11.6 vs. 5.3%, *p* < 0.001). This could be a potential confounder and was further adjusted by the three models in the conditional Cox regression analysis.

### Mortality and CV Events

#### Crude Incidence Rate

The median follow-up period was 59.6 months (25–75%, IQR: 22.8–108.9) after PSM. In patients without ESRD, the crude incidence rates of all-cause mortality were 80.2 and 80.3/1,000-person-years in patients who had mechanical and bioprosthetic valve replacement, respectively, and the crude incidence values of CV deaths were 43.4 and 41.4/1,000 person-years in patients who had mechanical and bioprosthetic valve replacement, respectively (Supplementary Table 4). In contrast, ESRD patients who underwent mechanical valve replacement had a higher rate of all-cause mortality (457.4/1,000 vs. 426.8/1,000 person-years) and CV death (262.0/1,000 vs. 218.7/1,000 person-years) than those who had bioprosthetic valve replacement (Table 2).

**Table 2:** Incidence rates and effect sizes of outcomes by valve replacement status in ESRD group.

**Outcomes**	**Variables**	**Total numbers**	**Event (%) / per 1,000 person-years**	**Models**	**Hazard ratios (95% CI)**	***P*** **value**
Total mortality	Patients with mechanical valve	456	412 (90.4%) / 457.4	0	1 (reference)	NA
				1		
				2		
				3		
	Patients with bioprosthetic valve	456	406 (89.0%) / 426.8	0	1.00 (0.95–1.06)	0.88
				1	0.98 (0.93–1.05)	0.55
				2	0.99 (0.93–1.05)	0.64
				3	0.88 (0.82–0.93)	<0.001
CV death	Patients with mechanical valve	456	236 (51.8%) / 262.0	0	1 (reference)	NA
				1		
				2		
				3		
	Patients with bioprosthetic valve	456	208 (45.6%) / 218.7	0	0.98 (0.90–1.06)	0.58
				1	0.96 (0.89–1.04)	0.29
				2	0.97 (0.89–1.05)	0.38
				3	0.83 (0.76–0.90)	<0.001

*CI, confidence interval; CV, cardiovascular; ESRD, end-stage renal disease; NA, not available*.

*Model 0: crude effect size by the two groups*.

*Model 1: adjusted effect by age, sex*.

*Model 2: adjusted effect by age, sex, total number of valves replaced, hypertension, diabetes mellitus, congestive heart failure, coronary artery diseases, and chronic obstructive pulmonary disease*.

*Model 3: adjusted effect by age, sex, total number of valves replaced, hypertension, diabetes mellitus, congestive heart failure, coronary artery diseases, chronic obstructive pulmonary disease, and medications (antiarrhythmic agents of Ia Ib, Ic, III, calcium channel blockers, angiotensin receptor blockers, statins, insulin, oral hypoglycemic agents)*.

#### Kaplan–Meier Survival Analysis

**Figure 2:**
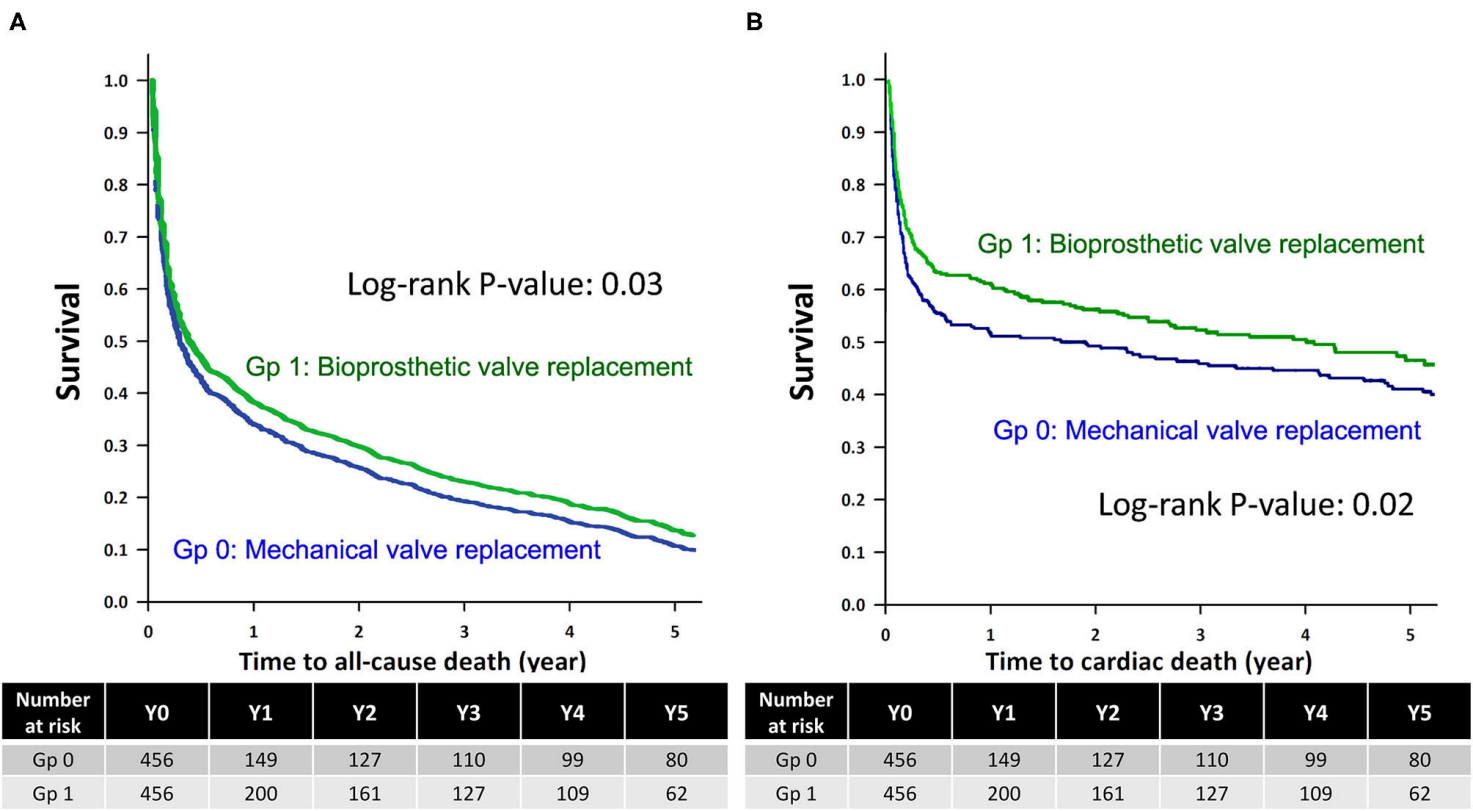
Kaplan–Meier survival analysis of the ESRD group. Kaplan-Meier survival analysis for **(A)** all-cause mortality and **(B)** cardiovascular deaths among ESRD patients who underwent mechanical valve replacement (Gp 0) and bioprosthetic valve replacement (Gp 1), with the statistical significance examined using the Log-rank test. ESRD, end-stage renal disease; Gp, group.

The Kaplan–Meier survival analysis revealed that the all-cause mortality and CV deaths were comparable between non-ESRD patients who underwent mechanical and bioprosthetic valve replacement during the 5-year follow-up (Log-rank test, *p* = 0.88 and 0.58, respectively; Supplementary Figures 1A,B). Meanwhile, ESRD patients who underwent mechanical valve replacement had higher rates of all-cause mortality and CV deaths than those who underwent bioprosthetic valve replacement after the 5-year follow-up (Log-rank test, *p* = 0.03 and 0.02, respectively; Figures 2A,B).

#### Multivariable Regression Analysis

After adjusting for the effects of age, sex, total number of valves replaced, underlying disease, and use of medications via a multivariable regression analysis, results showed a comparable future risk of all-cause mortality in non-ESRD patients who had mechanical and bioprosthetic valve replacement (*p* = 0.12, HR: 0.89, 95% CI: 0.77–1.03; Supplementary Table 4). However, non-ESRD patients who had bioprosthetic valve replacement had a significant decrease in the rate of CV deaths (*p* = 0.04, HR: 0.82, 95% CI: 0.67–0.99; Supplementary Table 4). In contrast, in ESRD patients, bioprosthetic valve replacement was significantly associated with a lower rate of all-cause mortality and CV deaths after adjusting for the confounding variables during the 5-year follow-up (*p* < 0.001, HR: 0.88, 95% CI: 0.82–0.93 and *p* < 0.001, HR: 0.83, 95% CI: 0.76–0.90, respectively; Table 2).

#### Short-Term Mortality and the Impact of Total Number of Valves Replaced

Regarding the perioperative and short-term mortality, the mortality rate occurring within 30 days after valve replacement was 19.2%, while the 1-year mortality rate was 63.6% in the present study.

In addition, the increasing total number of valves replaced was significantly associated with a higher rate of all-cause mortality and CV deaths both before and after adjusting for the confounding variables (in all models 0–3) during the 5-year follow-up in patients with ESRD (Supplementary Table 5).

## Discussion

### Major Findings

The current nationwide population-based study revealed the long-term outcome of ESRD and non-ESRD patients who underwent mechanical and bioprosthetic valve replacement. Firstly, despite the use of different types of valves for replacement, patients with ESRD before valvular surgery had a significantly worse outcome. Secondly, ESRD patients who had bioprosthetic valve replacement had a better long-term outcome in terms of all-cause mortality and CV deaths than those who underwent mechanical valve replacement.

### Outcome of Valve Replacement in ESRD Patients

Based on the 2019 Annual Report on Kidney Disease in Taiwan, the overall survival at 1, 3, 5, and 10 years of ESRD patients in Taiwan are 88.7, 69.2, 54.3, and 29.8%, respectively. The present study demonstrated remarkably poorer survival outcomes in ESRD patients with surgical valve replacement. Previous studies have shown that ESRD patients who underwent valve replacement have poor long-term outcomes (17–21). Based on a pooled analysis of eight studies, Altarabsheh et al. (17) have revealed that the overall survival at 2, 4, and 6 years among ESRD patients who had valve replacement was 58, 38, and 29%, respectively (median survival = 2.61 years) (17). Williams et al. (18) have found that only about half of dialysis patients younger than 65 years survived beyond 2 years after valve replacement (18). Moreover, Böning et al. have reported that the median survival time of ESRD patients after aortic valve replacement is 24.7 months (20). All the aforementioned findings were consistent with our results which demonstrated that ESRD patients had a worse prognosis than non-ESRD patients after bioprosthetic and mechanical valve replacement. Nevertheless, the perioperative and short-term mortality rate was higher than most of the previous reports. Firstly, the possible explanation is the remarkably older age of both groups in the present study (mean age 67.4 for the mechanical valve and 66.8 for the bioprosthetic valve group). In addition, unlike previous trials which were mainly based on evidence from single tertiary or referral centers, this nationwide population-based study might have more scientific rigor and external validity to reflect the real-world prognosis of the general population and clinical practice.

The cause of the poor prognosis among ESRD patients who had valve replacement are complex and multifactorial. Firstly, the disturbance in mineral metabolism and increased calcium load due to calcium-based phosphorus binders in the ESRD population can result in vascular calcification, which plays a key role in CV death (33). Calcium and phosphorus dysregulation may accelerate the calcification and structural destruction of valve bioprosthesis, which is mediated through a process of osteoblast-like differentiation on these structures (8–11). Moreover, several studies implicate that the host immune response is involved in a major pathogenesis of structural valve degeneration (34, 35). The persistent low-grade inflammation in patients with ESRD, which is associated with increased production and inadequate removal of pro-inflammatory cytokines, may accelerate the degeneration of bioprosthetic valves (36). On the other hand, a higher bleeding and thromboembolic risk has been observed in ESRD patients, (13, 14) particularly in those requiring anticoagulation therapy after mechanical valve replacement. Considering the abovementioned findings, the selection of valve for ESRD patients can be clinically challenging.

### Long-Term Outcome of Mechanical and Bioprosthetic Valve Replacement Among ESRD Patients

To the best of our knowledge, this is the first large-scale study that compared the outcome of mechanical and bioprosthetic valve replacement in ESRD patients using data from a nationwide population-based database. Of note, in ESRD patients in this study, bioprosthetic valve replacement was found to be significantly associated with a lower rate of all-cause mortality and CV deaths compared to replacement with mechanical valves. However, this result was not in accordance with that of previous studies showing that ESRD patients who had mechanical vs. bioprosthetic valve replacement had a similar survival time (26–30). The heterogeneous results could be explained by the differences in sample size, follow-up duration, use of medications, presence of comorbidities, advances in the development of prosthetic devices, and improvement in clinical care for dialysis patients between this and the other studies. Notably, several drugs showed a greater distribution in the bioprosthetic valve group, including class III antiarrhythmic drugs, angiotensin receptor blockers, and statins. It suggests the higher prevalence of concealed comorbidities in ESRD patients. It also explains why the benefit of bioprosthetic valves in the ESRD patients could finally be revealed after all the probable bias was minimized in Model 3.

Clinically, the selection of different valves for replacement is based on several factors, and there is no randomized study that compared the long-term outcomes of ESRD patients who underwent different types of valve replacement. Conventionally, ESRD patients were thought to experience early structural deterioration of the bioprosthetic valve due to disturbance in calcium homeostasis (8, 9). However, some studies have reported that this phenomenon is relatively rare due to the limited life expectancy of this population (4, 25). In contrast, some studies have revealed that ESRD patients with mechanical valves more commonly present with bleeding or thromboembolic events than structural deterioration of bioprosthetic valves (17, 24, 27). Patients with ESRD receiving mechanical valve replacement require warfarin for stroke prevention, which is usually associated with a higher risk of bleeding event, metastatic calcification, and catastrophic calciphylaxis (15, 16). The aforementioned complications could increase the periprocedural mortality, as shown in the present findings. However, future prospective studies must be conducted to validate the findings of the current study and to identify ESRD patients who are eligible for bioprosthetic valve replacement.

### Limitations

This study had some limitations. Firstly, this study is retrospective in nature, which might have caused inevitable bias. Given the entity of nationwide population-based study, additional information such as laboratory data, post-procedural complication and bleeding rate, causes of non-CV death, or causes of ESRD, cannot be retrieved from National Health Insurance Research Database. In addition, some detailed information was not available in the present study, including the presence of AF, concomitant CABG in valve replacement, specific cause of short-term mortality, or the survival data of ESRD patients without any valve replacement. However, our study can provide insight about clinical decision-making and can be used as a basis in further meta-analysis. Secondly, the baseline characteristics between the mechanical and bioprosthetic valve groups after PSM with potential covariates remained inconsistent. However, these potential confounders were all further adjusted by the three models in the conditional Cox regression analysis. We believe that the probable bias was minimized and assume that it's the reason why the benefit of bioprosthetic valves in the ESRD patients could finally be revealed. Third, diagnostic and procedure coding errors might exist. Nonetheless, the rate of coding error was supposed to be low because all data were double-checked by a professional coding team in each hospital before submission to the NHIRD. Fourth, this is a population-based study enrolling only people in Taiwan. It remains uncertain whether the findings are universal across various racial and ethnic groups in the world. In the end, based on the 2019 Annual Report on Kidney Disease in Taiwan, the majority of patients (around 90%) with ESRD received hemodialysis and <10% of ESRD patients undergoing peritoneal dialysis. Given the unbalanced distribution of hemodialysis vs. peritoneal dialysis and limited case numbers of study population, we didn't perform further analysis of the outcome between the above two groups. The further large cohort will be warranted for this investigation.

## Conclusion

Preoperative ESRD was associated with a significantly worse outcome in patients who had valve replacement. Patients with ESRD who underwent bioprosthetic valve replacement had significantly better long-term outcomes, including a lower rate of all-cause mortality and CV deaths. However, to shed light on clinical decision-making in this specific population, future prospective cohort studies based on independent databases are warranted to validate the present findings.

## Data Availability Statement

The datasets presented in this study can be found in online repositories. The names of the repository/repositories and accession number(s) can be found in the article/Supplementary Material.

## Ethics Statement

The studies involving human participants were reviewed and approved by Research Ethics Committee of the National Taiwan University Hospital. Written informed consent for participation was not required for this study in accordance with the national legislation and the institutional requirements.

## Author Contributions

All authors listed have made a substantial, direct, and intellectual contribution to the work and approved it for publication.

## Funding

This work was supported by the Center for Dynamical Biomarkers and Translational Medicine, Ministry of Science and Technology (Grant Numbers 107-2314-B-010-061-MY2, MOST 106-2314-B-075-006-MY3, MOST 106-2314-B-010-046-MY3, and MOST 106-2314-B-075-073-MY3), Research Foundation of Cardiovascular Medicine, Szu-Yuan Research Foundation of Internal Medicine (Grant Number 107-02-036), and Taipei Veterans General Hospital (Grant Numbers V108C-032, V108C-107, V109C-113, V109D48-001-MY2-1, C17-095, V106C-158, V106C-104, V107B-014, V107C-060, and V107C-054).

## Conflict of Interest

The authors declare that the research was conducted in the absence of any commercial or financial relationships that could be construed as a potential conflict of interest.

## Publisher's Note

All claims expressed in this article are solely those of the authors and do not necessarily represent those of their affiliated organizations, or those of the publisher, the editors and the reviewers. Any product that may be evaluated in this article, or claim that may be made by its manufacturer, is not guaranteed or endorsed by the publisher.

## Abbreviations

CI: confidence interval
CV: cardiovascular
ESRD: end-stage renal disease
HR: hazard ratio
IQR: interquartile range
NHIRD: National Health Insurance Research Database
PSM: propensity-score matching.
