# Supplementary material for: Long-Term Outcomes of Bioprosthetic or Mechanical Valve Replacement in End-Stage Renal Disease: A Nationwide Population-Based Retrospective Study
Source: Front Cardiovasc Med. 2021 Dec 17;8:745370. doi: 10.3389/fcvm.2021.745370 (PMC8718455; doi:10.3389/fcvm.2021.745370)
Supplement: Supplementary file 1 [file Data_Sheet_1.docx]

**Supplementary Table 1: Baseline characteristics of the original cohort**

| **Variable** | **Original cohort** | | |
| --- | --- | --- | --- |
|  | **Mechanical valve**  **(N=13537)** | **Bioprosthetic valve (N=5108)** | **P value** |
| **Age** | 58.4±14.6 | 64.7±13.4 | <0.001 |
| **Male gender** | 7947 (58.7%) | 2883 (56.4%) | 0.01 |
| **Valve location** |  |  |  |
| Aortic valve | 7497 (55.4%) | 2966 (58.1%) | 0.001 |
| Mitral valve | 7460 (55.1%) | 2500 (48.9%) | <0.001 |
| Tricuspid valve | 497 (3.7%) | 239 (4.7%) | 0.002 |
| Pulmonary valve | 38 (0.3%) | 49 (1%) | <0.001 |
| **Total number of valves replaced** | 1.14±0.36 | 1.13±0.35 | 0.002 |
| 1 | 11634 (85.9%) | 4481 (87.7%) | 0.001 |
| 2 | 1851 (13.7%) | 610 (11.9%) |  |
| 3 | 52 (0.4%) | 16 (0.31%) |  |
| 4 | 0 (0%) | 2 (0.04%) |  |
| **Comorbidities** |  |  |  |
| End-stage renal disease (%) | 1091 (8.1%) | 461 (9%) | 0.03 |
| Diabetes mellitus (%) | 579 (4.3%) | 223 (4.4%) | 0.79 |
| Hypertension (%) | 1582 (11.7%) | 596 (11.7%) | 0.97 |
| COPD (%) | 218 (1.6%) | 110 (2.2%) | 0.01 |
| Congestive heart failure (%) | 3028 (22.4%) | 1345 (26.3%) | <0.001 |
| Prior stroke (%) | 488 (3.6%) | 199 (3.9%) | 0.35 |
| Prior coronary artery disease (%) | 472 (3.5%) | 184 (3.6%) | 0.70 |
| Thyroid disease (%) | 31 (0.2%) | 11 (0.2%) | 0.86 |

Medicines were identified after propensity-score matching; therefore, we did not provide the medicine information of the original cohort.

COPD = chronic obstructive pulmonary disease.

| **Supplementary Table 2. Baseline characteristics of the propensity-score matched cohort** | | | |
| --- | --- | --- | --- |
| **Variables** | **After propensity-score matching** | | |
|  | **Mechanical valve**  **(N=5101)** | **Bioprosthetic valve (N=5101)** | **P value** |
| **Age** | 64.8±13.4 | 64.7±13.4 | 0.82 |
| **Male gender** | 2865 (56.2%) | 2882 (56.5%) | 0.73 |
| **Valve location** |  |  |  |
| Aortic valve | 2963 (58.1%) | 2963 (58.1%) | >0.99 |
| Mitral valve | 2606 (51.1%) | 2494 (48.9%) | 0.03 |
| Tricuspid valve | 146 (2.9%) | 237 (4.6%) | <0.001 |
| Pulmonary valve | 10 (0.2%) | 47 (0.9%) | <0.001 |
| **Total number of valves replaced** | 1.12±0.33 | 1.13±0.34 | 0.64 |
| 1 | 4488 (88%) | 4476 (87.7%) | 0.71 |
| 2 | 602 (11.8%) | 609 (11.9%) |  |
| 3 | 11 (0.2%) | 16 (0.31%) |  |
| 4 | 0 (0%) | 0 (0%) |  |
| **Comorbidities** |  |  |  |
| ESRD (%) | 456 (8.9%) | 456 (8.9%) | >0.99 |
| Diabetes mellitus (%) | 190 (3.7%) | 221 (4.3%) | 0.12 |
| Hypertension (%) | 582 (11.4%) | 594 (11.6%) | 0.71 |
| COPD (%) | 99 (1.9%) | 109 (2.1%) | 0.48 |
| CHF (%) | 1344 (26.3%) | 1345 (26.4%) | 0.98 |
| Prior stroke (%) | 169 (3.3%) | 197 (3.9%) | 0.14 |
| Prior CAD (%) | 257 (5%) | 184 (3.6%) | <0.001 |
| Thyroid disease (%) | 9 (0.2%) | 11 (0.2%) | 0.65 |
| **Pharmacotherapy *** | | | |
| AADs (%) | 2176 (42.7%) | 2367 (46.4%) | <0.001 |
| Class Ia | 90 (1.8%) | 114 (2.2%) | 0.09 |
| Class Ib | 537 (10.5%) | 566 (11.1%) | 0.36 |
| Class Ic | 343 (6.7%) | 372 (7.3%) | 0.26 |
| Class III | 1784 (35%) | 1981 (38.8%) | <0.001 |
| CCB (%) | 2394 (46.9%) | 2363 (46.3%) | 0.54 |
| ARB (%) | 3444 (67.5%) | 3618 (70.9%) | <0.001 |
| Statins (%) | 1941 (38.1%) | 2122 (41.6%) | <0.001 |
| Insulin (%) | 793 (15.5%) | 732 (14.4%) | 0.09 |
| OHA (%) | 1479 (29%) | 1487 (29.2%) | 0.86 |
| * used from baseline till the end of follow-up.  AAD = antiarrhythmic drugs; ARB = angiotensin receptor blockers; CAD = coronary artery disease; CCB = calcium channel blocker; CHF = congestive heart failure; COPD = chronic obstructive pulmonary disease; ESRD = end-stage renal disease; OHA = oral hypoglycemic agents. | | | |

| **Supplementary Table 3. Baseline characteristics of non-ESRD cohorts after propensity-score matching** |
| --- |

| **Variables** | **Non-ESRD group**  **(N=9290)** | | |
| --- | --- | --- | --- |
|  | **Mechanical valve**  **(N=4645)** | **Bioprosthetic valve**  **(N=4645)** | **P value** |
| **Age** | 64.6±13.6 | 64.5±13.6 | 0.96 |
| **Male gender** | 2625 (56.5%) | 2628 (56.6%) | 0.95 |
| **Valve location** |  |  |  |
| Aortic valve | 2721 (58.6%) | 2729 (58.8%) | 0.87 |
| Mitral valve | 2360 (50.8%) | 2240 (48.2%) | 0.01 |
| Tricuspid valve | 133 (2.9%) | 210 (4.5%) | <0.001 |
| Pulmonary valve | 10 (0.2%) | 44 (0.9%) | <0.001 |
| **Total number of valves replaced** | 1.12±0.34 | 1.12±0.34 | 0.98 |
| 1 | 4077 (87.8%) | 4081 (87.9%) | 0.82 |
| 2 | 557 (12%) | 550 (11.8%) |  |
| 3 | 11 (0.2%) | 14 (0.3%) |  |
| 4 | 0 (0%) | 0 (0%) |  |
| **Comorbidities** |  |  |  |
| ESRD (%) | 0 (0%) | 0 (0%) | >0.99 |
| Diabetes mellitus (%) | 174 (3.7%) | 199 (4.3%) | 0.19 |
| Hypertension (%) | 529 (11.4%) | 532 (11.5%) | 0.92 |
| COPD (%) | 95 (2%) | 105 (2.3%) | 0.48 |
| CHF (%) | 1226 (26.4%) | 1235 (26.6%) | 0.83 |
| Prior stroke (%) | 153 (3.3%) | 175 (3.8%) | 0.22 |
| Prior CAD (%) | 204 (4.4%) | 160 (3.4%) | 0.02 |
| Thyroid disease (%) | 9 (0.2%) | 11 (0.2%) | 0.65 |
| **Pharmacotherapy *** |  |  |  |
| AADs (%) | 2030 (43.7%) | 2192 (47.2%) | 0.001 |
| Class Ia | 83 (1.8%) | 106 (2.3%) | 0.09 |
| Class Ib | 502 (10.8%) | 524 (11.3%) | 0.47 |
| Class Ic | 319 (6.9%) | 343 (7.4%) | 0.33 |
| Class III | 1677 (36.1%) | 1843 (39.7%) | <0.001 |
| CCB (%) | 2191 (47.2%) | 2167 (46.7%) | 0.62 |
| ARB (%) | 3170 (68.2%) | 3296 (71%) | 0.004 |
| Statins (%) | 1757 (37.8%) | 1908 (41.1%) | 0.001 |
| Insulin (%) | 659 (14.2%) | 576 (12.4%) | 0.01 |
| OHA (%) | 1311 (28.2%) | 1295 (27.9%) | 0.71 |
| * used from baseline till the end of follow-up.  AAD = antiarrhythmic drugs; ARB = angiotensin receptor blockers; CAD = coronary artery disease; CCB = calcium channel blocker; CHF = congestive heart failure; COPD = chronic obstructive pulmonary disease; ESRD = end-stage renal disease; OHA = oral hypoglycemic agents. | | | |

| **Supplementary Table 4. Incidence rates and effect sizes of outcomes by valve replacement status in non-ESRD group** | | | | | | |
| --- | --- | --- | --- | --- | --- | --- |
| **Outcomes** | **Variables** | **Total numbers** | **Event (%) / per 1000 person-years** | **Models** | **Hazard ratios (95% CI)** | **P value** |
| **Total mortality** | **Patients with mechanical valve** | 4645 | 2559 (55.1%) /80.2 | 0 | 1 (reference) | NA |
|  |  |  |  | 1 |  |  |
|  |  |  |  | 2 |  |  |
|  |  |  |  | 3 |  |  |
|  | **Patients with bioprosthetic valve** | 4645 | 2209 (47.6%) /80.3 | 0 | 0.91 (0.79-1.26) | 0.19 |
|  |  |  |  | 1 | 0.91 (0.79-1.04) | 0.16 |
|  |  |  |  | 2 | 0.93 (0.81-1.06) | 0.28 |
|  |  |  |  | 3 | 0.89 (0.77-1.03) | 0.12 |
| **CV deaths** | **Patients with mechanical valve** | 4645 | 1348 (29.0%) /43.4 | 0 | 1 (reference) | NA |
|  |  |  |  | 1 |  |  |
|  |  |  |  | 2 |  |  |
|  |  |  |  | 3 |  |  |
|  | **Patients with bioprosthetic valve** | 4645 | 1140 (24.5%) /41.4 | 0 | 0.81 (0.67-0.98) | 0.03 |
|  |  |  |  | 1 | 0.80 (0.66-0.96) | 0.02 |
|  |  |  |  | 2 | 0.82 (0.68-0.99) | 0.04 |
|  |  |  |  | 3 | 0.82 (0.67-0.99) | 0.04 |
| CI = confidence interval; CV = cardiovascular; ESRD = end-stage renal disease; NA = not available  Model 0: crude effect size by the two groups;  Model 1: adjusted effect by age, sex;  Model 2: adjusted effect by age, sex, total number of valves replaced, hypertension, diabetes mellitus, congestive heart failure, coronary artery diseases, and chronic obstructive pulmonary disease;  Model 3: adjusted effect by age, sex, total number of valves replaced, hypertension, diabetes mellitus, congestive heart failure, coronary artery diseases, chronic obstructive pulmonary disease, and medications (antiarrhythmic agents of Ia Ib, Ic, III, calcium channel blockers, angiotensin receptor blockers, statins, insulin, oral hypoglycemic agents). | | | | | | |

| **Supplementary Table 5. Effect sizes of outcomes by total number of valves replaced in ESRD group** | | | | |
| --- | --- | --- | --- | --- |
| **Outcomes** | **Variables** | **Models** | **Hazard ratios (95% CI)** | **P-value** |
| **Total mortality** | **Total number of valves replaced** | 0 | 1.19 (1.09-1.29) | <0.001 |
|  |  | 1 | 1.19 (1.09-1.29) | <0.001 |
|  |  | 2 | 1.18 (1.08-1.28) | <0.001 |
|  |  | 3 | 1.19 (1.10-1.30) | <0.001 |
| **CV deaths** |  | 0 | 1.29 (1.15-1.44) | <0.001 |
|  |  | 1 | 1.29 (1.15-1.44) | <0.001 |
|  |  | 2 | 1.28 (1.14-1.43) | <0.001 |
|  |  | 3 | 1.28 (1.15-1.44) | <0.001 |

CI = confidence interval; CV = cardiovascular; ESRD = end-stage renal disease

Model 0: crude effect size by the four groups;

Model 1: adjusted effect by age, sex;

Model 2: adjusted effect by age, sex, hypertension, diabetes mellitus, congestive heart failure, coronary artery diseases, and chronic obstructive pulmonary disease;

Model 3: adjusted effect by age, sex, hypertension, diabetes mellitus, congestive heart failure, coronary artery diseases, chronic obstructive pulmonary disease, and medications (Antiarrhythmic agents of Ia Ib, Ic, III, calcium channel blockers, angiotensin receptor blockers, statins, insulin, oral hypoglycemic agents).

Supplementary Figure 1

**
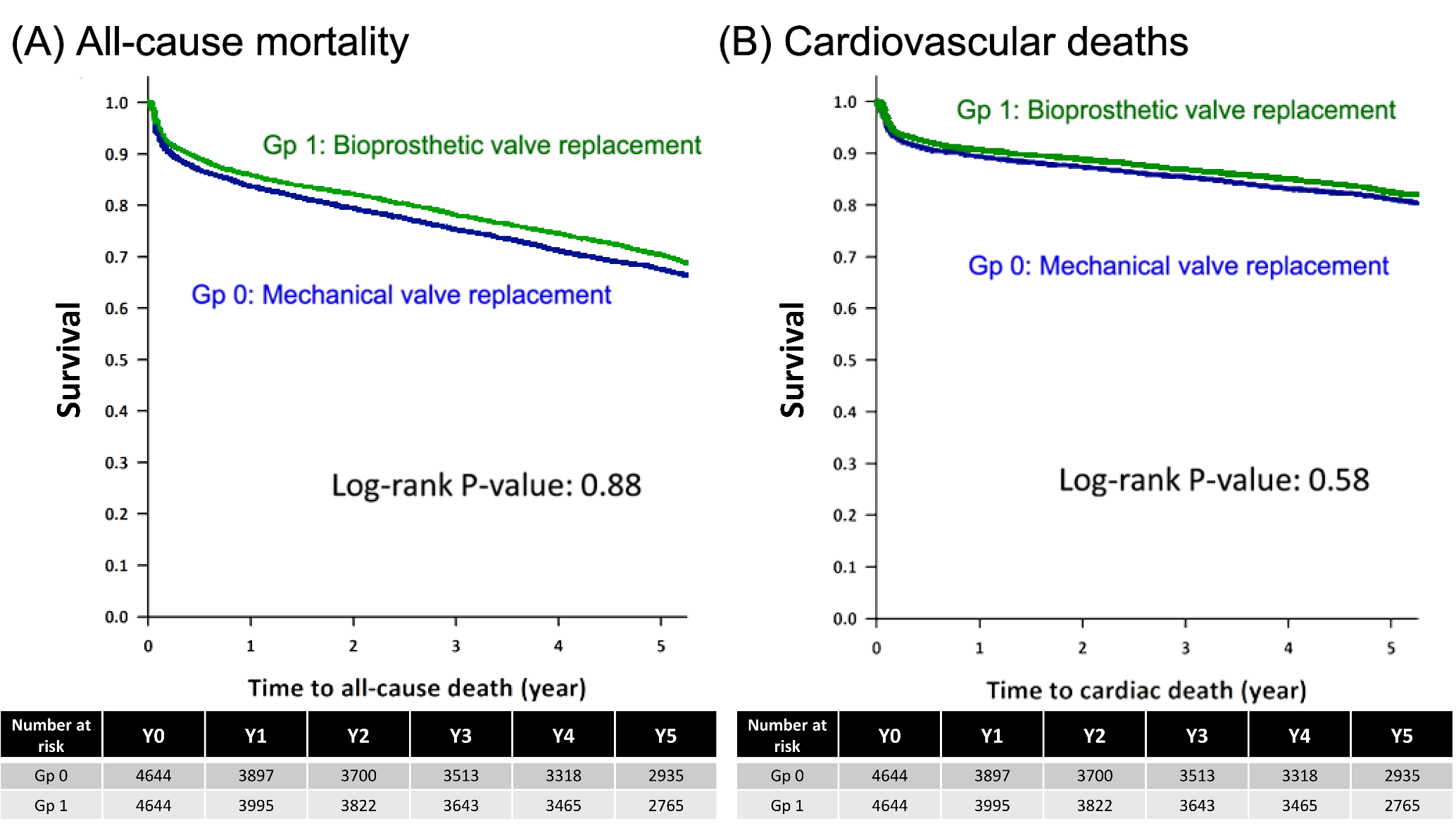
**

**Figure Legends**

**Supplementary Figure 1.** Kaplan–Meier survival analysis of the non-ESRD group.

Kaplan-Meier survival analysis for **(A)** all-cause mortality and **(B)** cardiovascular deaths, among non-ESRD patients who underwent mechanical valve replacement (Gp 0) and bioprosthetic valve replacement (Gp 1), with the statistical significance examined using the log-rank test.

ESRD = end-stage renal disease; Gp = group.

Order codes in the *National Health Insurance Research Database*

- **Hemodialysis**: 580001C, 58019C, 58020C, 58021C, 58022C, 58023C, 58024C, 58025C, and 58029C
- **Peritoneal dialysis**: 580002C, 58009A, 58009B, 58010A, 58010B, 58011A, 58011AB, 58011B, 58011C, 58012A, 28012B, 58017B, 58017C, 58026C, and 58028C
- **Other types of dialysis**: 58018C, 58027C, and 58030B
- **Kidney transplantation**: 76020A, 76020B, 97416K, 97417A, and 97418B
